# Accidental Sewing Pin Ingestion by a Tailor: A Case Report and Literature Review

**DOI:** 10.3390/medicina59091566

**Published:** 2023-08-29

**Authors:** Stefan Stojkovic, Milica Bjelakovic, Milica Stojkovic Lalosevic, Milos Stulic, Nina Pejic, Nemanja Radivojevic, Nemanja Stojkovic, Jelena Martinov Nestorov, Djordje Culafic

**Affiliations:** 1 Clinic for Gastroenterology and Hepatology, University Clinical Center of Serbia, 11000 Belgrade, Serbia; 2 Clinic for Gastroenterology and Hepatology, University Clinical Center of Nis, 18000 Nis, Serbia; 3 Faculty of Medicine, University of Belgrade, 11000 Belgrade, Serbia; 4 Clinic for Otorhinolaryngology and Maxillofacial Surgery, University Clinical Center of Serbia, 11000 Belgrade, Serbia; 5 Department of Cardiology, University Clinical Hospital Center “Dr. Dragisa Misovic-Dedinje”, 11000 Belgrade, Serbia

**Keywords:** foreign body ingestion, sewing pin, stomach, endoscopy, tailor

## Abstract

Foreign body ingestion is a frequently encountered emergency in healthcare institutions. It mostly affects pediatric populations, although it can also affect adults with developmental delays, those with psychiatric diseases, drug abusers, and prisoners. Endoscopy is a diagnostic and treatment method for suspected foreign body ingestion. In this article, we discuss a 45-year-old tailor who swallowed a sewing pin while at work. The abdominal X-ray showed a needle-shaped metal shadow in the stomach region. During an upper endoscopy, it was discovered that a sewing pin with a sharp edge was stuck in the pylorus. The sewing pin was extracted endoscopically, and the patient was discharged the same day in good condition. Since the estimated risk of complications of foreign body ingestion in the adult population is about 35%, and the most common complications include impaction, laceration, bleeding, or perforation of the gastrointestinal wall, endoscopic or surgical removal is necessary. This also emphasizes the importance of a careful endoscopic evaluation of some at-risk occupations for foreign body ingestion with or without gastrointestinal complaints.

## 1. Introduction

Foreign body (FB) ingestion is a frequently encountered emergency in healthcare institutions. Although the majority of patients with FB ingestions are found in the pediatric population, these emergencies also affect adults. In particular, impaction with meat or fish bones while eating is the most common cause of FB ingestion in adults. Patients with developmental delays, psychiatric diseases, abusers of illicit drugs or alcohol, and prisoners are recognized as high-risk groups for accidental or intentional ingestion of FB [1]. While most of the FB pass spontaneously through the gastrointestinal tract, in 10% to 20% of patients, endoscopic extraction is necessary, and in less than 1% of patients, surgical intervention is required [2]. Swallowed objects, especially sharp ones, may cause serious complications during their passage through the digestive tube [3,4]. Symptoms may vary depending on the type of FB ingested and the time interval until diagnosis.

Swallowed sharp foreign objects cause 15–35% of intestinal perforations and should be removed, especially if found in the esophagus or stomach [5]. Endoscopy is a well-known diagnostic and treatment modality for suspected FB ingestion since it reduces the need for surgery and makes it possible to diagnose other diseases at the same time [6,7].

Here we report a case of accidental sewing pin ingestion by a tailor.

## 2. Case Presentation

**Figure 1:**
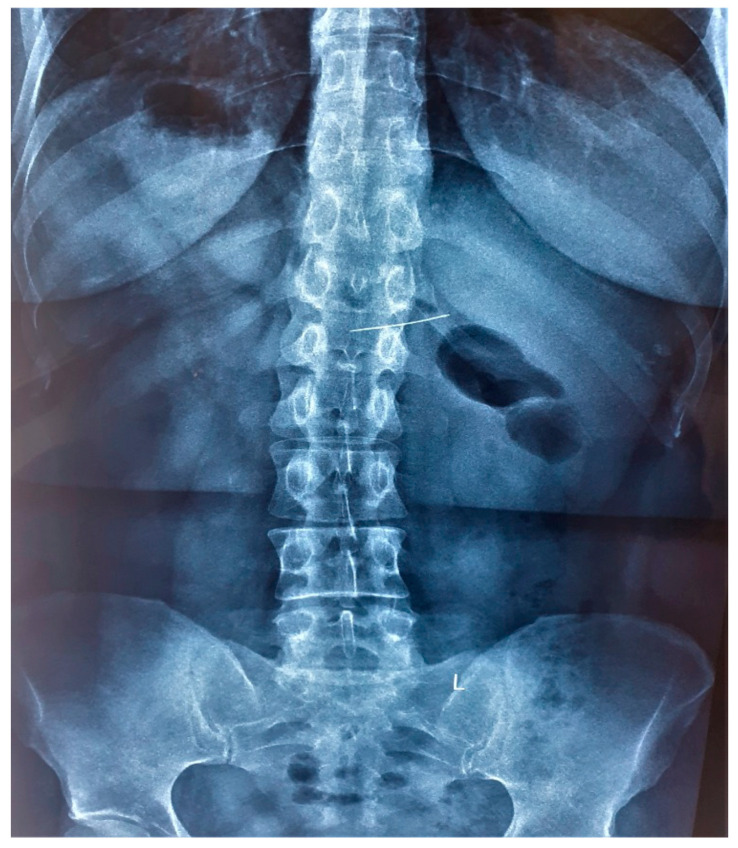
A metal shadow in the stomach region shown in a plain abdominal X-ray image.

**Figure 2:**
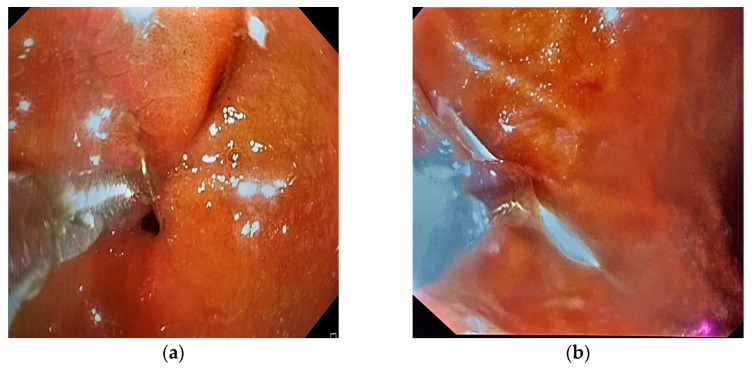
Upper endoscopy findings. (**a**) A sewing pin stuck in the pylorus with surrounding tissue reaction. (**b**) An alligator jaw forceps holding the sewing pin in the stomach.

**Figure 3:**
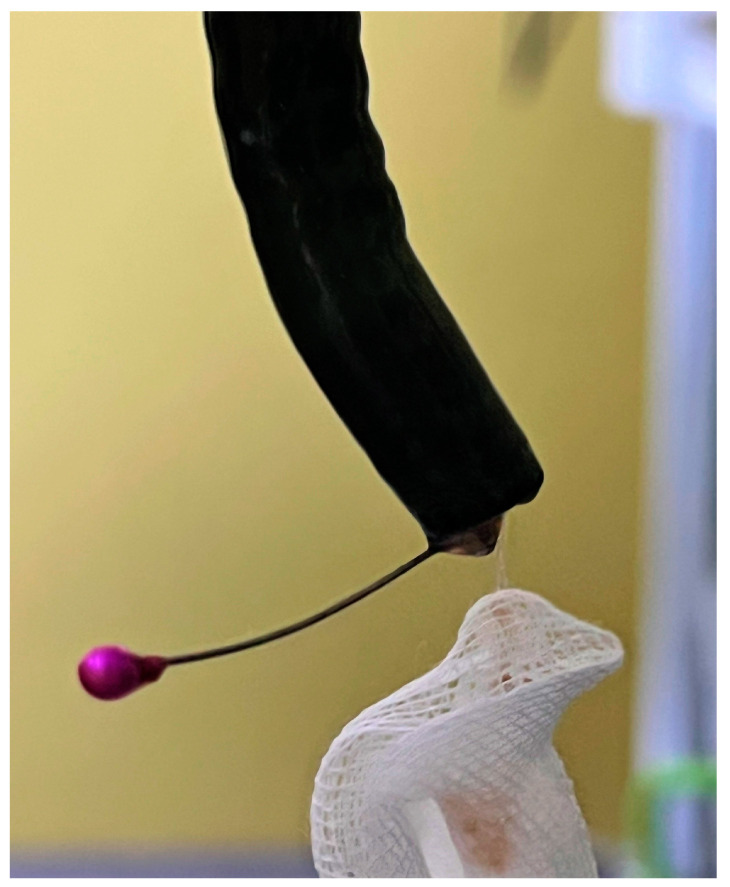
An extracted sewing pin.

A 45-year-old female tailor was admitted to the emergency department due to the possible swallowing of a sewing pin. She reported that she had been holding several sewing pins with her lips during work the day before when she accidentally coughed. She did not know if she had swallowed a pin because she could not remember the exact number of pins she was holding. Later that day, she felt slight discomfort in her upper abdominal parts. Past medical and surgical history was not specific. Abdominal palpation revealed a slight pain in the epigastrium. Other physical and laboratory findings were unremarkable. The patient was immediately referred to a plain abdominal X-ray, which showed a needle-shaped metal shadow, approximately 4 cm long, in the stomach region (shown in Figure 1). Thereafter, an urgent upper endoscopy was performed, and a needle was seen in the pyloric part of the stomach. The sharp point of the pin was stuck in the pylorus with surrounding tissue reaction, while the blunt, rounded, purple-colored pinhead was in the duodenal bulb (shown in Figure 2a). Alligator jaw forceps were used, and the pin was gripped near its center, between the pinhead and the tip. Firstly, a gentle push was applied towards the bulbus to disengage the pin from the pylorus. After that, the pin was slowly pulled through the pylorus in the antrum. In the antrum grasp of alligator forceps was relocated from the pin’s center towards the tip (shown in Figure 2b). After a firm grip was obtained, the pin was gently extracted with the tip of the forceps about a centimeter away from the endoscope tip to provide direct visualization during extubation. Protective overtubes were not used because of the endoscopist’s certainty of the forceps grip, direct visualization at all times, and the fact that the pinhead was rounded and not supposed to damage the tissues by contact (shown in Figure 3). After the procedure, a control endoscopy was performed, and since there was no mucosal laceration on the esophagus or stomach, the patient was discharged in good condition.

## 3. Discussion

The management of patients with FB ingestion requires different diagnostic and therapeutic approaches due to the specificity of each individual case. The lack of specific complaints that are clearly related to the localization of the FB and the need for timely identification, as well as the treatment of complications, require the coordinated work of radiologists, endoscopists, and surgeons [4,8].

In patients presenting with FB ingestion, symptoms may vary from asymptomatic or mildly uncomfortable to dramatic and life-threatening. Most patients have symptoms, and they vary depending on the type and location of the FB [9]. Usually, patients after FB ingestion present with sensations of FB, dysphagia, chest or abdominal pain, nausea, or vomiting [10]. In symptomatic patients, FB are more likely to be located in the esophagus and pharynx. By contrast, in asymptomatic patients, FBs are more frequently located in the stomach or duodenum [9,11]. Moreover, sharp objects are more likely to cause symptoms than blunt FBs. Furthermore, the absence of symptoms leads to a late diagnosis and the development of complications [12]. Therefore, all patients suspected of FB ingestion should undergo radiologic and/or laryngological examination to exclude oropharyngeal location [10]. Since our patient had very mild symptoms and was not sure that she had swallowed a needle, the decision to perform an X-ray and endoscopy proved to be very useful. Endoscopic treatment can be challenging because the estimated risk of complications is as high as 35%, especially if the FB is not removed at the right time [13]. The success rate of endoscopic FB removal is 95%, while adverse events are rare and include mucosal laceration, bleeding, infection, perforation, or aspiration [9,14]. According to the European Society of Gastrointestinal Endoscopy, emergency upper endoscopy is recommended within 6 h in cases of esophageal obstruction or sharp objects in the esophagus and within 24 h in cases of other FBs. An endoscopic examination is also recommended after removing the FB [3]. A study by Liu et al. showed that a time interval of 6 h can reduce all complications, while a time interval between 6 and 24 h can reduce major complications but not all of them [15].

Complications of sharp object ingestion include impaction, laceration, bleeding, or perforation of the gastrointestinal wall, especially in the anatomically narrow parts of the gastrointestinal tract, such as the upper and lower esophageal sphincters, the pylorus, the ileocecal valve, and, in rare cases, the appendiceal lumen [4]. In our case, the needle was impacted in the pylorus, so the risk of serious complications was high. Thus, endoscopic treatment was performed immediately with great caution to avoid injury to the gastrointestinal wall and potential aspiration. Endoscopic management of sharp FB extraction includes various techniques and instruments. These techniques involve using an overtube or a transparent cup, or fashioning a protective hood [4]. Shishido et al. [16] described the successful extraction of a sewing needle that was grasped by biopsy forceps and withdrawn together with the endoscope through a flexible overtube placed in the duodenum. Instead, in our case, we were able to remove the pin safely only by grasping the tip of the needle using endoscopic forceps, whereas a smooth pinhead should not be able to cause complications. Similarly, Costa et al. [17] successfully removed thirteen needles from the gastric body and one from the duodenum by grasping the sharp end of the needle with continuous suction into the working channel of the endoscope. Thus, endoscopic extraction of such objects is easier than that of objects with two pointed sides because there is low risk of injury to the contralateral wall. Therefore, a detailed description of the swallowed FB and an X-ray are necessary [18]. The possibility of spontaneous passage is affected by several factors, including the size, shape, and composition of the ingested FB. The duration of ingestion and the age of the patient also play a significant role in the management of these patients [1].

Objects less than 2.5 cm in diameter can pass spontaneously [19], but cases of later complications due to their migration have been described by some authors. Sharp objects may reach the liver or pancreas in 1% of cases by penetrating the small intestine or stomach wall. Considering the high possibility of unhindered passage through the gastrointestinal tract, some authors still advocate conservative treatment in the case of sharp or pointed FB, regardless of the potential risk. As a result, Bazabih and Getu [5] only used follow-up and serial radiological exams to successfully treat a 23-year-old male who ingested a metal nail. In contrast, Dal et al. [20] described an accidentally swallowed sewing needle in a 23-year-old female that was revealed starting from the posterior of the small curvature of the stomach and reaching the head and body of the pancreas. This patient required laparoscopic surgery for the removal of the FB. In the literature, the most commonly reported complications of an unextracted FB are intestinal perforation, abscess formation, fistulae, and appendicitis [21,22]. In order to prevent complications, it is recommended that sharp FBs be extracted whenever possible, and in the event of unsuccessful extraction, clinical follow-up and daily radiographs are necessary. If it is observed that the FB does not progress within three days after ingestion, surgical intervention should be considered [3].

Several observational studies have shown that up to 80% of patients who ingest FBs are children [21]. In adults, most FB ingestion or food bolus impaction occurs accidentally during eating. It is important to emphasize that underlying esophageal pathology, such as stricture or malignancy, is found in more than 75% of patients with food bolus impaction [23,24]. The rest of the patients marked as having a high risk of FB ingestion are the geriatric population, adults with psychiatric illnesses, and individuals under the influence of drugs or alcohol. A special group consists of patients who benefit from FB ingestion, such as prisoners or drug couriers [3]. The most frequently found ingestions of sharp FBs include meat bones, toothpicks, needles, safety pins, and dental appliances [25]. The type of FB that is swallowed is influenced by various factors, including dietary habits, behavior, and religion. For example, several studies have shown that the accidental ingestion of date pits while eating is very common in China, while ingestion of hijab pins is common among Muslim women [26,27]. Hijab pins provide a tiny but significant risk of catastrophic sequelae, especially in cases of needle impaction lasting longer than a few months, according to a large cohort study by Yogev et al. that included 208 patients who swallowed a hijab needle. [28] A CT scan should be done on patients who have ingested a needle in the last several weeks or months to rule out infectious or mechanical issues [29]. However, there is little information in the literature about the ingestion of FB in specific professions.

We performed a literature search in the PubMed database (Medline) using the keywords “foreign body ingestion”, “sewing pin”, and “tailor’’ and identified only two related publications. Ha et al. [30] reported the case of a 34-year-old tailor who swallowed a sewing needle and, after an unsuccessful colonoscopy, underwent a laparoscopic appendectomy. Another publication by Espin et al. [31] describes the case of a tailor who was diagnosed with colonic perforation after swallowing a needle and was treated surgically with a laparoscopic approach. To our knowledge, this is the first case report found in the literature of an endoscopic retraction of a sewing pin in a patient who is at occupational risk for ingestion—a tailor. This further emphasizes that patients with specific occupations should be noted as high-risk groups for FB ingestion.

## 4. Conclusions

An ingested sharp FB may cause serious complications in the gastrointestinal tract, such as perforation or migration into other organs. An endoscopy ought to be performed even when FB ingestion is suspected. The presence of a sharp FB requires endoscopic or surgical removal. This therapeutic method must be performed with extreme caution to avoid injury to the gastrointestinal wall and aspiration. A CT scan should be done if the patient presents later than two to three weeks after ingesting. Even seasoned tailors who hold pins with their lips while working, though this is a widespread technique throughout the world, run the risk of ingesting them.

## Data Availability

All information is publicly available, and data regarding this particular patient can be obtained from the corresponding author upon request.
